# Prevalence of multimorbid degenerative lumbar spinal stenosis with knee or hip osteoarthritis: a systematic review and meta-analysis

**DOI:** 10.1186/s12891-022-05104-3

**Published:** 2022-02-24

**Authors:** James J. Young, Rikke Krüger Jensen, Jan Hartvigsen, Ewa M. Roos, Carlo Ammendolia, Carsten Bogh Juhl

**Affiliations:** 1 Center for Muscle and Joint Health, University of Southern Denmark, Campusvej 55, 5230 Odense M, Denmark; 2 Research Division, Canadian Memorial Chiropractic College, 6100 Leslie Street, Toronto, Canada; 3 Centre for Muscle and Joint Health, Department of Sports Science and Clinical Biomechanics, University of Southern Denmark, 55 Campusvej, DK-5230 Odense M, Denmark; 4 Chiropractic Knowledge Hub, Odense M, Denmark; 5 Rebecca MacDonald Centre for Arthritis and Autoimmune Diseases, Mount Sinai Hospital, Toronto, Canada; 6 Institute for Health Policy, Management and Evaluation, University of Toronto, Toronto, Canada; 7 Department of Physiotherapy and Occupational Therapy, Copenhagen University Hospital, Herlev and Gentofte, Copenhagen, Denmark

**Keywords:** Lumbar spinal stenosis, Knee osteoarthritis, Hip osteoarthritis, Multimorbidity, Prevalence

## Abstract

**Background:**

Musculoskeletal multimorbidity is common and coexisting lumbar spinal stenosis (LSS) with knee or hip osteoarthritis (OA) has been reported. The aim of this review was to report the prevalence of multimorbid degenerative LSS with knee or hip OA based on clinical and/or imaging case definitions.

**Methods:**

Literature searches were performed in MEDLINE, EMBASE, CENTRAL, and CINAHL up to May 2021. Studies involving adults with cross-sectional data to estimate the prevalence of co-occurring LSS with knee or hip OA were included. Study selection, data extraction, and risk of bias assessment were performed independently by two reviewers. Results were stratified according to index and comorbid condition, and by case definitions (imaging, clinical, and combined).

**Results:**

Ten studies from five countries out of 3891 citations met the inclusion criteria. Sample sizes ranged from 44 to 2,857,999 (median 230) and the mean age in the included studies range from 61 to 73 years (median 66 years). All studies were from secondary care or mixed settings. Nine studies used a combined definition of LSS and one used a clinical definition. Imaging, clinical, and combined case definitions of knee and hip OA were used. The prevalence of multimorbid LSS and knee or hip OA ranged from 0 to 54%, depending on the specified index condition and case definitions used. Six studies each provided prevalence data for index LSS and comorbid knee OA (prevalence range: 5 to 41%) and comorbid hip OA (prevalence range: 2 to 35%). Two studies provided prevalence data for index knee OA and comorbid LSS (prevalence range 17 to 54%). No studies reporting prevalence data for index hip OA and comorbid LSS were found. Few studies used comparable case definitions and all but one study were rated as high risk of bias.

**Conclusions:**

There is evidence that multimorbid LSS with knee or hip OA occurs in people (0 to 54%), although results are based on studies with high risk of bias and surgical populations. Variability in LSS and OA case definitions limit the comparability of studies and prevalence estimates should therefore be interpreted with caution.

**Review registration:**

PROSPERO (CRD42020177759).

**Supplementary Information:**

The online version contains supplementary material available at 10.1186/s12891-022-05104-3.

## Background

Multimorbidity (the co-existence of two or more chronic conditions) is an increasing health challenge as global populations continue to age [1–3]. Chronic musculoskeletal comorbidities are frequently reported as part of the multimorbid chronic disease profile [4] and increase the risk of developing other non-communicable diseases [5]. Multimorbidity must be considered in care models for musculoskeletal health [6] and older adults [7].

The prevalence of degenerative lumbar spinal stenosis (LSS) with knee or hip osteoarthritis (OA) – three of the most common chronic musculoskeletal conditions in the aging population – continue to rise [8–10]. Similar to knee and hip OA, LSS is most often a result of degenerative changes [11] and these diseases may co-exist in people with either index condition [12]. Supporting this hypothesis, a recent analysis from the Johnston County OA Project found 44% and 34% of people with imaging findings associated with lumbar spine OA also had image-defined knee and hip OA, respectively [13]. Clinical evidence also suggests lower extremity arthritis is common in people with LSS undergoing surgery [14, 15].

In both LSS and OA, radiological and clinical case definitions are used in prevalence estimates, and results differ according to the case definition employed [9, 16]. No gold-standard case definition exists for either LSS or knee and hip OA, but definitions where both clinical and imaging findings are combined may be preferred since symptoms experienced by people with LSS and knee and hip OA often do not relate to the degree of structural change observed via imaging [11, 17].

Irrespective of criteria used, co-occurring disease is likely important in the clinical decision-making process. Multimorbidity in general is associated with a worse quality of life and future functional decline [18–20]. Musculoskeletal-specific multimorbidity is also associated with decreased health status metrics [21–23], particularly true in both low back pain [24] and multi-joint OA [25, 26]. In people with LSS, the presence of comorbidities (including lower extremity arthritis) is associated with worse preoperative function [14], and comorbid knee and hip OA are associated with increased odds of poorer postoperative function [15]. Finally, people with LSS are less likely to attain a meaningful improvement following surgery when they also report additional symptomatic joints, including the knee and hip [27].

A comprehensive summary of available literature is needed to determine how frequently LSS and knee and hip OA coexist as a first step in improving clinical decision-making. The overall aim of this review was to report the prevalence of multimorbid degenerative LSS with knee or hip OA based on clinical and/or imaging case definitions.

## Methods

The protocol for this review has been published [28] and registered in PROSPERO (CRD42020177759). This review was conducted according to the guidelines from the Cochrane Collaboration [29] and reported according to the Preferred Reporting Items for Systematic Reviews and Meta-Analysis (PRISMA) statement [30] (Additional file 1).

### Objectives

This review had four objectives:

1. To estimate the prevalence of index LSS with comorbid knee OA based on imaging, clinical, and combined case definitions.
2. To estimate the prevalence of index knee OA with comorbid LSS based on imaging, clinical, and combined case definitions.
3. To estimate the prevalence of index LSS with comorbid hip OA based on imaging, clinical, and combined case definitions.
4. To estimate the prevalence of index hip OA with comorbid LSS based on imaging, clinical, and combined case definitions.

#### Case definitions

All case definitions for degenerative LSS and knee and hip OA were included and were broadly classified into three categories as imaging; clinical; and combined diagnoses encompassing both imaging and clinical diagnoses. The following diagnoses and clinical manifestations were included based upon study eligibility criteria: All imaging diagnoses based on radiographic, magnetic resonance imaging, or computerized tomography for the lumbar spine, knee, and hip; clinical diagnoses based on signs and symptoms of LSS, knee OA, and hip OA; all clinical manifestations of LSS (neurogenic claudication, radicular type, and mixed types) as they represent central, lateral, and combined central and lateral canal stenosis [31]. Patient self-reported diagnoses and medical chart reviews, including International Classification of Disease codes, were considered to be clinical diagnoses for both LSS and OA, unless explicitly stated otherwise. We considered surgical samples for LSS and/or OA (without explicit case definitions) to be combined diagnoses as it is unlikely that individuals would be offered surgery based upon imaging or clinical findings alone. For example, if a study included participants undergoing surgery for knee OA who were also evaluated for LSS by imaging, knee OA was designated as the index condition and assigned a combined case definition, whereas LSS was designated as the comorbid condition and assigned an imaging case definition.

#### Search strategy

The literature search was performed on May 03, 2021 in the following databases with no publication date or language limitation: MEDLINE, EMBASE, CENTRAL, and CINAHL. The search strategy was developed with a health sciences librarian based upon previous Cochrane reviews on LSS [32] and knee [33] and hip OA [34]. Search terms related to LBP were included in the LSS search concept to ensure potentially relevant literature was captured. The search was developed in MEDLINE and adapted to the other databases (Additional file 2).

Forward citation tracking of included studies was performed in Web of Science and reference lists were searched for additional relevant studies. The reference list from a recent review on LSS prevalence [9] was searched for relevant studies. PROSPERO was searched for relevant ongoing or completed systematic reviews. Additionally, scientific abstracts presented at the International Forum for Back and Neck Pain Research in Primary Care and the Osteoarthritis Research Society International World Congress between 2018 and 2020 were reviewed to identify publications potentially relevant to this review. Finally, LSS content experts known to the author team were also contacted to identify any known publications relevant to our research question.

#### Eligibility criteria

Studies were considered for inclusion if they: (1) were cross-sectional, cohort or case-control studies, or randomized controlled trials; (2) included adults 18 years or older; (3) assessed the prevalence of co-occurring LSS with knee and/or hip OA or present sufficient cross-sectional data for estimating the prevalence (number of participants with LSS, number of participants with knee and/or hip OA, and total number of participants); and (4) were full-text papers published in English in peer-reviewed journals. Studies were excluded if they: (1) included individuals with low back, knee, or hip pain due to other origins (e.g., fracture, tumour, inflammatory disease, infection, lumbar disc herniation); (2) were laboratory or cadaveric studies; (3) were conference abstracts; (4) included congenital or non-degenerative forms of LSS, without separate data on degenerative LSS; or (5) provided aggregate prevalence data for knee and hip OA separately.

#### Study selection

Records identified in the search strategy were screened using a two-stage process by two reviewers (JJY and RKJ) using a sensitive screening approach where any title or abstract mentioning any of the following were moved to full-text review: (1) LSS in isolation; (2) knee or hip OA and comorbidities; (3) knee or hip OA and LBP; or (4) prevalence of multiple musculoskeletal conditions. This approach was taken to mitigate the exclusion of relevant studies due to lack of reporting of information relevant to this review in the abstract. First, reviewers independently screened titles and abstracts against the eligibility criteria. Discrepancies were resolved through discussion.

Next, full-text versions of the remaining potentially relevant studies were independently screened. Discrepancies were resolved through discussion or independent review by a third reviewer (CBJ) when necessary. Reasons for exclusion of full-text articles were recorded. All references returned in the database search were managed using Endnote X9 (Clarivate Analytics, Philadelphia, USA) and Covidence systematic review software (Veritas Health Innovation, Melbourne, Australia). Absolute agreement and the Kappa coefficient [35] was calculated for both stages of screening using the Covidence feature.

#### Data extraction

The following information was extracted from all included papers by two independent reviewers (JJY and RKJ) using a standardized extraction form: first author, publication year, country, study design, population (LSS, knee OA, or hip OA), inclusion criteria, study setting, age, sex, case definitions of LSS and knee and/or hip OA, numerator and denominator for prevalence calculation, and items used in the risk of bias assessment.

#### Risk of bias assessment

Two reviewers (JJY and RKJ) independently assessed the risk of bias in all included studies using a modified version of the Risk of Bias Tool for Prevalence Studies [36]. Modifications made to the tool for the purposes of this study have been described in detail in the review protocol [28] (Additional file 3). In summary, we removed item 5 from the original tool as we assumed both clinical and imaging information can only be collected directly from participants. Further, items 6 and 7 (acceptability of case definition and validity/reliability of study instruments) were split into two questions to allow for individual ratings of LSS and OA definitions and measurement properties. Finally, we included an additional response option of “irrelevant” for item 9 because studies using imaging definitions are not impacted by recall bias. The modifications for this study followed the approach taken in two recent systematic reviews using the same tool [9, 24].

Individual items were rated as “Yes” for low risk of bias or “No” for high risk of bias or if there was insufficient information presented for adequate judgement. No specific quantitative thresholds were used to judge the overall risk of bias. Rather, a judgement on the internal and external validity taking into consideration the responses to each item on the modified tool was used to determine the overall risk of bias (low, moderate, high) by the two reviewers.

#### Data management and synthesis

Pooled prevalence estimates with 95% CI for each combination of LSS with knee or hip OA (based on case definitions) was performed using a random effects model. Due to the limited number of studies using comparable case definitions for LSS and knee and/or hip OA, only pooled estimates for combined LSS and co-occurring (i) clinical knee OA (ii) imaging knee OA, (iii) clinical hip OA, and (iv) imaging hip OA were calculated and are summarized. One study reporting a prevalence of 0% was artificially given a numerator of 0.001. The low number of studies used in each of the pooled estimates prevented the calculation of heterogeneity statistics and no pre-planned meta-regression analyses [28] were performed. All statistical analyses were performed in Stata 16.1 (StataCorp LLC, College Station, USA).

## Results

### Study selection

**Fig. 1:**
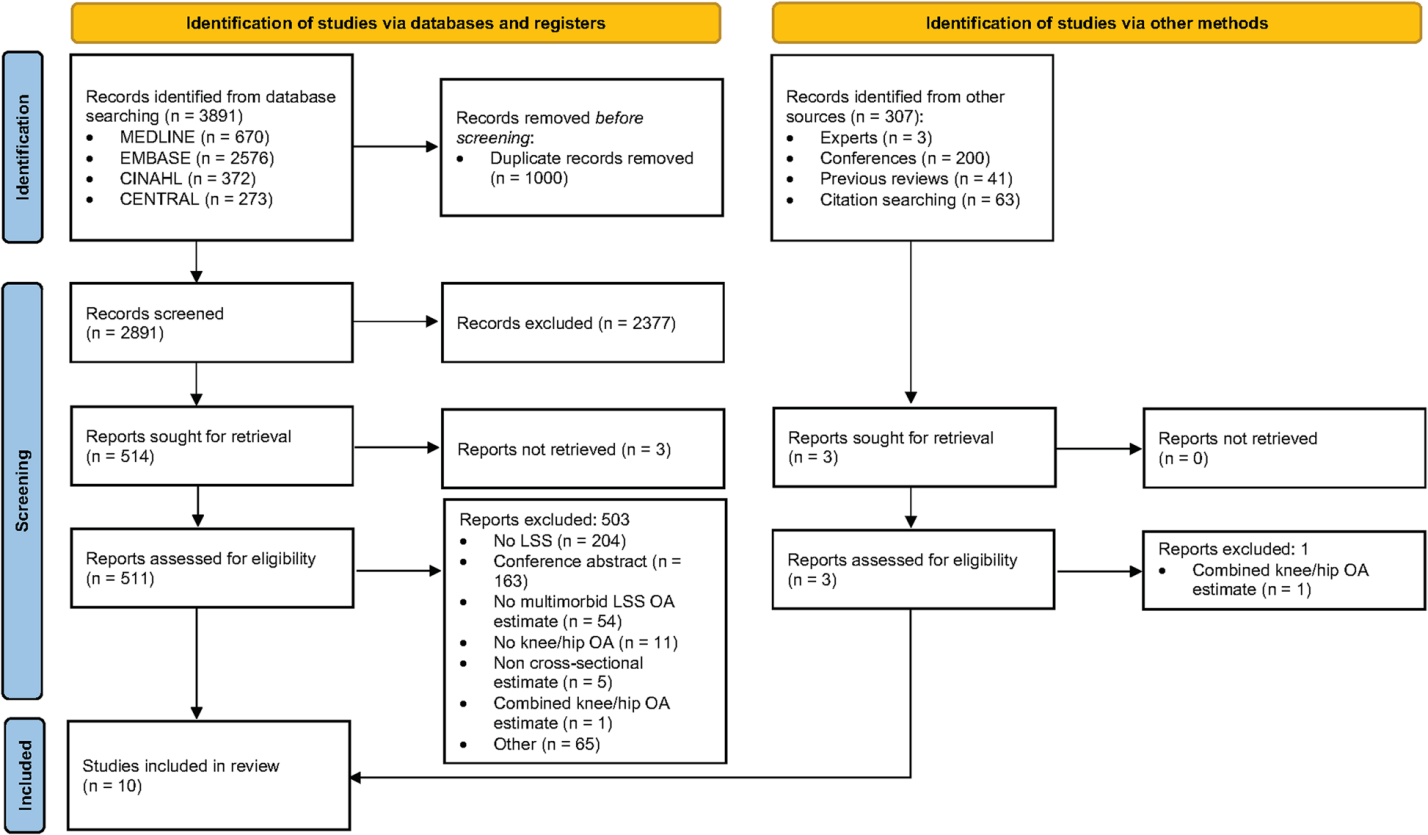
PRISMA flow chart of literature search and study selection

The database search identified a total of 3891 citations with 307 additional citations identified through other sources (Additional file 1). After removal of duplicates, titles and abstracts of the remaining 2891 records were screened and 517 articles were deemed relevant for full-text screening. Absolute agreement between reviewers was 90% with a Kappa of 0.62. After full-text screening, ten articles met the eligibility criteria for this review [37–46]. Two excluded articles provided prevalence data on LSS with comorbid knee and hip OA, but were excluded because separate estimates for knee and hip OA were not provided [47, 48]. Absolute agreement for full text screening was 98% with a Kappa of 0.68. The study selection process is presented in Fig. 1.

### Study characteristics and results

No included studies had an objective of estimating the prevalence of multimorbid LSS with knee and/or hip OA. Sample sizes ranged from 44 to 2,857,999 (median 230) with a median age of 66 years (range 61 - 73).

### LSS and comorbid knee OA

Six studies estimating the prevalence of index LSS with comorbid knee OA were included [37, 39–41, 43, 45] (Table 1). Half of the studies were from Korea (*n*=3) [39–41], with one study each from Japan [43], USA [45], and Switzerland [37]. Study designs were cross-sectional (*n*=2) [40, 41], cohort (*n*=2) [37, 39], case-control (*n*=1) [43], and randomized controlled trial (*n*=1) [45]. All studies were from secondary care settings with the exception of one study that included a mixed sample [45]. Sample sizes ranged from 44 to 641 (median 183). The proportion of females included ranged from 52 to 100% and the mean age ranged from 61 to 73 years (median 68). A combined case definition (imaging and clinical findings) was used in all studies for the index LSS condition. Combined (*n*=1), clinical (*n*=2), and imaging (*n*=3) case definitions of comorbid knee OA were used (Table 1).

**Table 1:** Characteristics of included studies for multimorbid lumbar spinal stenosis with knee osteoarthritis

Author, year	Country	Study design	Index condition: setting	Mean age (SD)	Sex (% female)	LSS case definition	LSS sample size	Knee OA case definition	Knee OA sample size	Multimorbid prevalence
**LSS with comorbid knee OA**										
Kim, 2008a [39]	Korea	Cohort	LSS: secondary care surgical setting	61 (NR)	100%	Combined: undergoing surgery	44	Imaging: K/L grade 2-4	19	43%
Kim, 2008b [40]	Korea	Cross-sectional	LSS: secondary care surgical setting	67 (7.0)	100%	Combined: neurogenic claudication + MRI	67	Imaging: K/L grade 2-4	27	40%
Lee, 2012 [41]	Korea	Cross-sectional	LSS: secondary care surgical setting	66 (8.4)	100%	Combined: neurogenic claudication + MRI	106	Imaging: K/L grade 2-4	44	42%
Schneider, 2019 [45]	USA	RCT	LSS: mixed general population/primary/secondary care	72 (7.8)	52%	Combined: clinical examination + MRI/CT	259	Clinical: ACR criteria	82	32%
Burgstaller, 2020 [37]	Switzerland	Cohort	LSS: eight secondary care centers	73 (8.5)	52%	Combined: neurogenic claudication + MRI/CT	601	Clinical: self-reported	133	22%
Ozaki, 2020 [43]	Japan	Case-control	LSS: secondary care surgical setting	KOA group only: 69 (8.3)	KOA group only: 71%	Combined: neurogenic claudication + MRI/myelography	641	Combined: medical history + K/L grade 2-4	32	5%
**Knee OA with comorbid LSS**										
Cho, 2019 [38]	Korea	Cross-sectional	Knee OA: national health insurance database claims	64 (9.8)	62%	Clinical: ICD-10 codes	479,311	Combined: ICD-10 codes for diagnosis and imaging	2,857,999	17%
Londhe, 2020 [42]	India	Cohort	Knee OA: secondary care surgical setting	66 (14.8)	82%	Combined: symptoms + MRI/CT	108	Combined: undergoing surgery	200	54%

*LSS* lumbar spinal stenosis, *KOA *knee osteoarthritis, *NR *not reported, *Combined *clinical and imaging findings of LSS or KOA, *ACR *American College of Rheumatology, *MRI *magnetic resonance imaging, *CT* computed tomography, *K/L* Kellgren-Lawrence, *ICD-10* International Classification of Diseases 10th revision

**Fig. 2:**
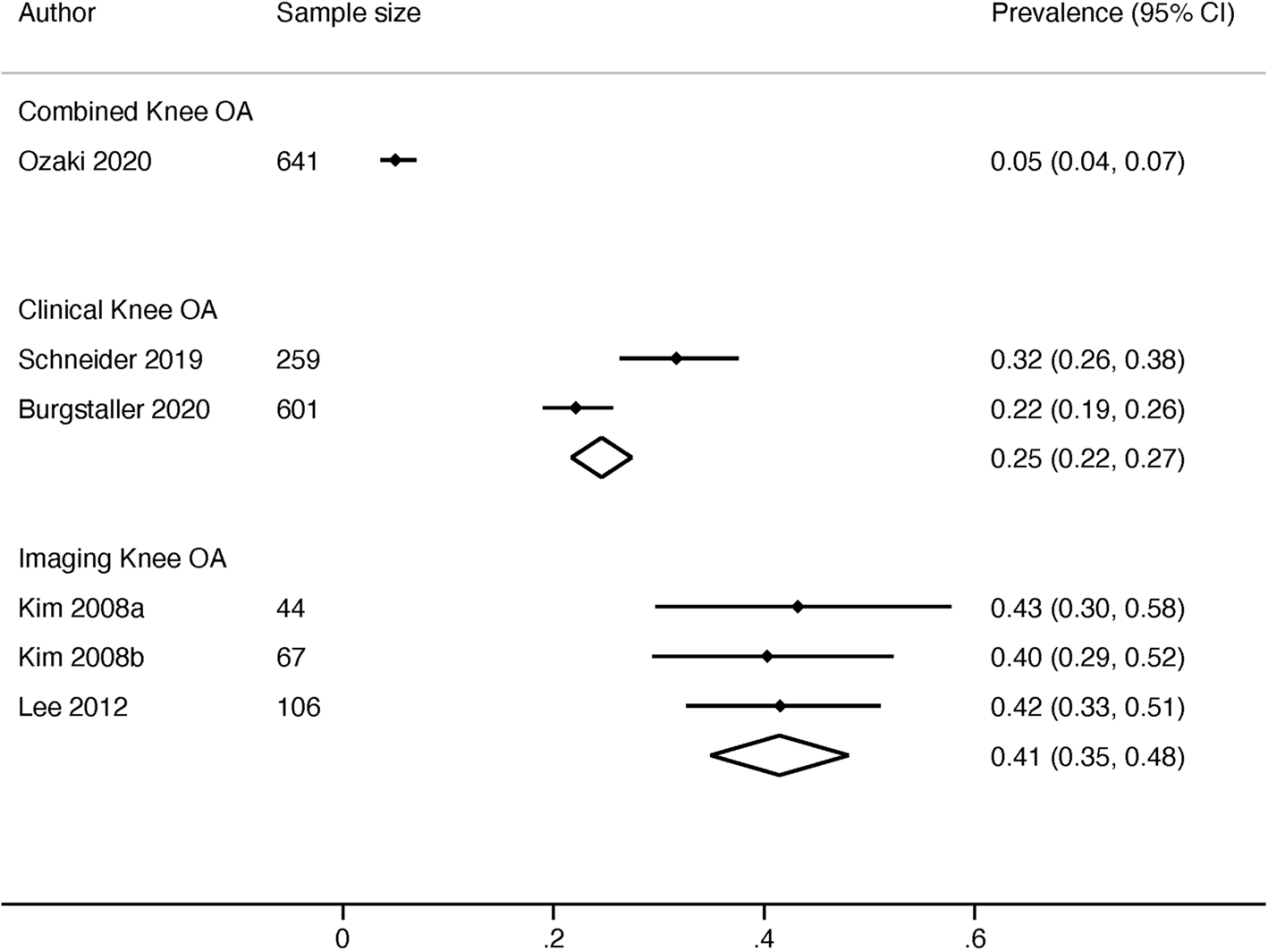
Forest plot of results for combined lumbar spinal stenosis with comorbid knee osteoarthritis

The single study (*n*=641) using a combined definition of LSS with a combined definition of knee OA found a prevalence of 5% (95% CI 4-7%) [43]. The pooled prevalence in two studies (*n*=860) using combined definitions of LSS with clinical definitions of knee OA was 25% (95% CI 22-27%) [37, 45]. The pooled prevalence in three studies (*n*=217) using combined definitions of LSS and imaging definitions of knee OA was 41% (95% CI 35-48%) [39–41] (Fig. 2).

### Knee OA and comorbid LSS

Two studies estimating the prevalence of index knee OA with comorbid LSS were included [38, 42] (Table 1). One study each was from Korea [38] and India [42] with one study using a cross-sectional design [38] and one a cohort [42]. One study used a sample from a secondary care [42] and the other from a national health insurance database [38]. Sample sizes were 200 and 2,857,999, the proportion of females was 62 and 82%, respectively and the mean ages were 64 and 66 years. Both studies used a combined case definition for the index knee OA condition. Combined (*n*=1) and clinical (*n*=1) case definitions of comorbid LSS were utilized (Table 1).

It was not possible to pool prevalence estimates of knee OA with comorbid LSS. The single study (*n*=200) using a combined definition of knee OA and a combined definition of LSS reported a prevalence of 54% (95% CI 47-61%) [42] whereas the other (*n*=2,857,999) using a combined definition of knee OA and a clinical definition of LSS reported a prevalence of 17% (95% CI 17-17%) [38].

### LSS and comorbid hip OA

Six studies estimating the prevalence of index LSS with comorbid hip OA were included [37, 39, 41, 44–46] (Table 2). Studies were performed in Korea (*n=2*) [39, 41], USA (*n*=2) [45, 46], Japan [44], and Switzerland [37]. Study designs were primarily cohort (*n*=4) [37, 39, 44, 46] with one cross-sectional study [41] and one randomized controlled trial [45]. All included studies enrolled LSS patients from secondary care settings, except one which included LSS patients from mixed settings [45]. Study sample size ranged from 44 to 601 participants (median 226). The proportion of females ranged from 3 to 100% and mean age from 61 to 73 years (median 66; one study age not reported). All studies used a combined case definition for the index LSS condition. Combined (*n*=1), clinical (*n*=2), and imaging (*n*=3) case definitions of comorbid hip OA were utilized (Table 2).

**Table 2:** Characteristics of included studies for multimorbid lumbar spinal stenosis with hip osteoarthritis

Author, year	Country	Study design	Index condition: setting	Mean age (SD)	Sex (% female)	LSS case definition	LSS sample size	Hip OA case definition	Hip OA sample size	Multimorbid prevalence
**LSS with comorbid hip OA**										
Kim, 2008a [39]	Korea	Cohort	LSS: secondary care surgical setting	61 (NR)	100%	Combined: undergoing surgery	44	Imaging: K/L grade 2-4	0	0%
Saito, 2012 [44]	Japan	Cohort	LSS: secondary care surgical setting	NR	NR	Combined: low back and leg pain + MRI/myelography/CT	420	Imaging: not described	15	4%
Lee, 2012 [41]	Korea	Cross-sectional	LSS: secondary care surgical setting	66 (8.4)	100%	Combined: neurogenic claudication + MRI	106	Imaging: K/L grade 2-4	4	4%
Schneider, 2019 [45]	USA	RCT	LSS: mixed general population/primary/secondary care	72 (7.8)	52%	Combined: clinical examination + MRI/CT	259	Clinical: ACR criteria	43	17%
Burgstaller, 2020 [37]	Switzerland	Cohort	LSS: eight secondary care centers	73(8.5)	52%	Combined: neurogenic claudication + MRI/CT	601	Clinical: self-reported	102	17%
Weiner, 2021 [46]	USA	Cohort	LSS: military veterans secondary care surgical setting	a	3%	Combined: neurogenic claudication + MRI	193	Combined: ACR clinical + radiographic criteria	68	35%

*LSS* lumbar spinal stenosis, *OA *osteoarthritis, *NR *not reported, *ACR *American College of Rheumatology, *MRI *magnetic resonance imaging, CT = computed tomography, *K/L* Kellgren-Lawrence

**Fig. 3:**
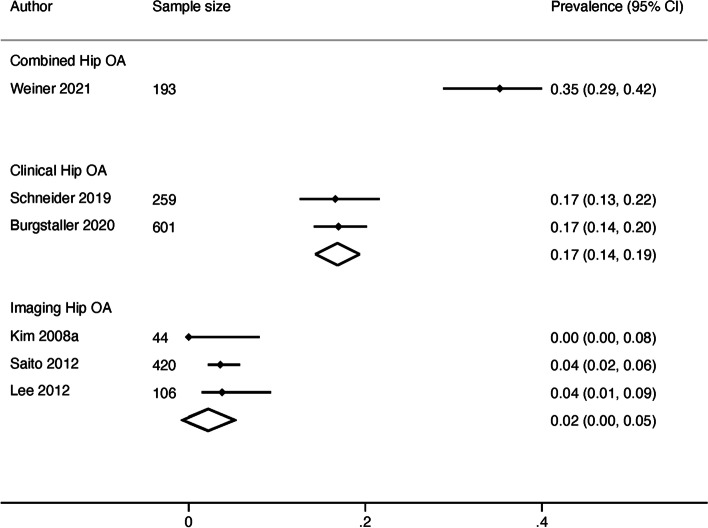
Forest plot of results for combined lumbar spinal stenosis with comorbid hip osteoarthritis

The single study (*n*=193) using a combined case definition of LSS with a combined definition of hip OA was 35% (95% CI 29-42%) [46]. The pooled prevalence in two studies (*n*=860) using combined definitions of LSS and clinical definitions of hip OA was 17% (95% CI 14-19%) [37, 45]. The pooled prevalence in three studies (*n*=570) using combined definitions of LSS and imaging definitions of hip OA was 2% (95% CI 0-5%) [39, 41, 44] (Fig. 3).

### Hip OA and comorbid LSS

No studies estimating the prevalence of hip OA with comorbid LSS met our eligibility criteria.

### Risk of bias

All included studies were judged as having a high risk of bias, with the exception of one study that had a moderate risk of bias [38] (Table 3). External validity items were generally rated as high risk of bias, as all but one study [38] did not include random samples representative of the target population and did not provide information on study response rates. Internal validity was also generally rated as having a high risk of bias. Most studies used an acceptable case definition for LSS and knee or hip OA, but with inadequate validity and reliability of the case definitions.

**Table 3:** Risk of bias assessment of included studies

	External validity				Internal validity							
Author, year	Study popula-tion represen-tative of target popula-tion	Sampling frame represen-tative of target popula-tion	Random selection or census	Non-response bias minimal	Accept-able definition of lumbar spinal stenosis	Accept-able definition of knee/hip osteo-arthritis	LSS instrument was valid and reliable	OA instrument was valid and reliable	Same mode of data collection for all subjects	Length of the shortest preva-lence period for parameter of interest was appro-priate	Numer-ator and denomin-ator for parameter of interest was appro-priate	Overall risk of bias
Kim, 2008a [39]	No	No	No	No	Yes	Yes	No	Yes	Yes	Yes	Yes	High
Kim, 2008b [40]	No	No	No	No	Yes	Yes	Yes	Yes	Yes	Yes	Yes	High
Saito, 2012 [44]	No	No	No	No	Yes	Yes	No	No	No	Yes	Yes	High
Lee, 2012 [41]	No	No	No	No	Yes	Yes	No	Yes	Yes	Yes	Yes	High
Cho, 2019 [38]	Yes	Yes	Yes	Yes	Yes	Yes	No	Yes	Yes	Yes	Yes	Moderate
Schneider, 2019 [45]	No	No	No	No	Yes	Yes	No	No	No	Yes	Yes	High
Burgstaller, 2020 [37]	No	No	No	No	Yes	Yes	No	No	No	Yes	Yes	High
Ozaki, 2020 [43]	No	No	No	No	Yes	Yes	No	Yes	No	No	Yes	High
Londhe, 2020 [42]	No	No	No	No	No	Yes	No	Yes	No	Yes	Yes	High
Weiner, 2021 [46]	No	No	No	Yes	Yes	Yes	No	No	Yes	Yes	Yes	High

Yes = low risk of bias, No = high risk of bias, High = high risk of bias (further research is very likely to have an important impact on our confidence in the estimate and is likely to change the estimate), Moderate = moderate risk of bias (further research is likely to have an important impact on our confidence in the estimate and may change the estimate), Low = low risk of bias (further research is very unlikely to change our confidence in the estimate). This risk of bias tool was adapted from the original Risk of Bias Tool for Prevalence Studies in low back pain research created by Hoy et al., [36] for specific use in people with lumbar spinal stenosis and knee or hip osteoarthritis

## Discussion

The prevalence of multimorbid LSS and knee or hip OA ranged from 0 to 54%, depending on the specified index condition and case definitions used. The majority of multimorbid prevalence estimates are derived from samples of participants with an index condition of LSS and comorbid knee or hip OA. Few studies used the same combination of case definitions for the index and comorbid condition, but the comparable studies did show similar prevalence estimates. All but one included study were high risk of bias. Included prevalence estimates were primarily derived from surgical samples. However, in presenting the first pooled data and prevalence estimates for multimorbid LSS with knee or hip OA this study may be of value to those planning and delivering musculoskeletal care in the expanding older population and may help to guide future prevalence studies.

### Prevalence estimates

Estimates for LSS with comorbid knee OA exhibited an increase in prevalence moving from combined to clinical to imaging knee OA definitions. The opposite pattern was observed in estimates of LSS and comorbid hip OA, where combined hip OA definitions had the highest prevalence and imaging the lowest. This pattern is counterintuitive because imaging findings are a prerequisite of combined (imaging findings plus clinical symptoms) case definitions. Many people with imaging evidence of OA are clinically asymptomatic [16] and therefore we would expect the prevalence of combined imaging plus clinical definitions to be less prevalent than imaging-only definitions, as observed in the LSS with comorbid knee OA estimates.

An insufficient number of studies on index knee or hip OA and comorbid LSS prevented us from comparing prevalence patterns. However, in the two studies providing data on knee OA (combined case definitions) and comorbid LSS, a greater prevalence was observed for combined comorbid LSS than clinical comorbid LSS (54% vs. 17%), suggesting a similarly counterintuitive pattern.

A meta-analysis by Pereira et al., [16] found the prevalence of radiographic hip OA was 15% versus 11% for symptomatic (combined radiographic and clinical definition) hip OA, which is different than the findings of our review (2% imaging; 35% combined). Conversely, the findings of our pooled analysis on LSS with comorbid knee OA follow the observed pattern found by Pereira et al., [16], where radiographic knee OA prevalence estimates were greater than symptomatic prevalence estimates (32% versus 21%, respectively). In one study providing data on the prevalence of comorbid LSS in a knee OA sample primarily from secondary care settings, the prevalence of clinically-defined LSS [17% (95% CI 17-17%)] [38] was lower than that observed in a recent meta-analysis of prevalence estimates of clinically-defined LSS in secondary care samples [29% (95% CI 22-36%)] and mixed primary and secondary care samples [39% (95% 38-39%)] [9]. The second study reporting the prevalence of comorbid LSS in a secondary care knee OA sample [54% (95% CI 47-61%)] [42] was much higher than reported in the LSS prevalence review.

### Risk of bias

We were unable to assess the risk of bias impact on our prevalence estimates since all but one included study were rated as high risk of bias. The high risk of bias ratings are mainly a result of no studies having the aim of investigating the multimorbid prevalence. Therefore, the included studies may have adequate design for their primary purpose but suffer from a high risk of bias when appraised for the purpose of estimating prevalence. However, we did adapt a single-condition risk of bias tool [36] for the purposes of this study. Our ratings may therefore lack validity, but little guidance on risk of bias in multimorbidity prevalence studies is available. For example, multimorbidity prevalence reviews have not evaluated risk of bias [22] or have used reporting checklists as a surrogate measure [49]. To overcome this issue, we further modified a risk of bias tool used in a recent review on the prevalence of low back pain and co-occurring musculoskeletal pain sites [24] and in a review on the prevalence of LSS [9]. Future studies with low risk of bias might provide more clarity on the seemingly opposite prevalence patterns observed in this review. Further, consensus standards for studies of multimorbidity prevalence, and musculoskeletal-multimorbidity specifically, are first needed.

### Defining LSS and OA

Different multimorbidity prevalence patterns may exist when using alternate case definitions for LSS. The multimorbidity prevalence pattern may also differ in samples where knee or hip OA is the index condition. Only two studies included data allowing for prevalence estimates of knee OA and comorbid LSS [38, 42] and we found no studies of hip OA and comorbid LSS. We were also unable to explore differences in prevalence estimates for varying clinical presentations of LSS, such as neurogenic claudication, radicular pain, or radiculopathy [31] as well as the impact differing levels of participant or clinical characteristics such as disease severity or stage, as this information was not consistently reported in the included studies.

No consensus on the exact features that define cases of LSS exists, whether based on imaging [50, 51] or clinical definitions. [52–54] The prevalence of multimorbid LSS and knee or hip OA relying on imaging-only case definitions may not be the best proxy, as imaging findings do not reliably represent the symptomatic experience of people with OA [17] or LSS [11]. In our review, only one included study specified the imaging findings considered to represent LSS [40]. The defining features of imaging-based knee and hip OA were more consistently reported in the included studies, but one study including imaging findings in the case definition did not describe the specific criteria used [44].

Combined clinical and imaging definitions likely represent the most reliable definition of LSS, however, when reported, variability existed in the exact imaging and clinical criteria. Many studies used a neurogenic claudication plus imaging findings on MRI, CT, or myelogram, but exact definitions were not reported. Likewise, studies with people undergoing surgery for LSS did not always report the surgical eligibility criteria.

### Influence of health care setting

We were unable to assess the impact of health care setting on our results. Prevalence estimates of LSS [9], OA [16], and multimorbidity in general [2] differ according to the population of interest. Jensen et al. found that [9] the prevalence of LSS ranged from 11% in the general population to 29% in secondary care settings. All but two studies included in our review enrolled participants from secondary care settings [38, 45]. Although insurance claim data from all levels of health care was included, 73% of participants in the study by Cho et al., [38] received care in secondary care orthopedic and internal medicine departments. The second study by Schneider et al., [45] used a mixed sample of people from the general population, primary care, and secondary care, without providing data on the proportion of participants enrolled from each. Understanding the differences in prevalence estimates in diverse health care settings may help guide management priorities [55, 56].

### Implications for future studies

Future studies should attempt to clarify the role chronic musculoskeletal conditions like LSS and OA play within the larger multimorbid burden faced by individuals and health systems. Musculoskeletal conditions increase one’s risk of developing other non-communicable diseases [5], evidenced by the number of comorbidities in people with OA [57, 58] and LSS [14, 59]. For example, Cho et al., [38] found Korean people with knee OA had comorbid: LSS (17%), hypertension (45%), diabetes (29%), liver disease (20%), depression (11%), and other chronic conditions. Therefore, the prevalence of multimorbid LSS and knee and/or hip OA represents only a small portion of the larger impact experienced by those living with multiple chronic conditions [4, 60]. Additionally, we only examined the prevalence of multimorbid LSS and knee or hip OA independently, but co-occurring patterns including both knee, hips, and the lumbar spine may be more informative. Future studies should also pay particular attention to how multimorbidity is defined and measured, as variable case definitions (including symptoms and/or risk factors) alter prevalence estimates for LSS [9], OA [16], and multimorbidity in general [2, 60, 61].

### Limitations

The main limitation of this review is the lack of original studies with a primary objective matching our review question. As a result, included studies were at a high risk of bias, but it is unclear what effect this may have on prevalence estimates. It was also difficult to assess the eligibility of articles based on titles and abstracts alone. We employed an overly sensitive approach to study screening, where any article mentioning “lumbar spinal stenosis” alone, “knee or hip OA and comorbidities”, or multiple musculoskeletal conditions was screened in full text. Despite this approach, two included studies [37, 45] were not captured in the search. It is possible other published articles contain relevant data to this review but were not identified. Echoing recommendations from the wider multimorbidity literature [61], future reviews on this topic would benefit from the inclusion of a “multimorbidity” indexing term in electronic databases.

Heterogeneity in case definitions for LSS and lack of studies establishing a valid and reliable method of LSS assessment also limits our findings. Two studies included case definitions such as self-report of conditions and diagnostic codes, which were not easily classified into our pre-specified case definition framework. More available relevant studies would allow for sensitivity analyses to evaluate the assumptions made when classifying these case definitions. Finally, debate exists on the most suitable method of measuring multimorbidity. The method of simply counting the presence of conditions is supported in previous literature [2], but may be limited in comparison to definitions including symptoms and risk factors [61]. Future studies investigating multimorbid LSS and knee and hip OA would be greatly improved with the standardization of case definitions for LSS, OA, and multimorbidity.

## Conclusions

This review has summarized the evidence for the co-occurrence of LSS with knee or hip OA. There is evidence that LSS with knee or hip OA appears to be common, but that estimates are uncertain as the prevalence ranged from 0 to 54% depending on the joint assessed and case definition. The variability in case definitions used for both LSS and OA, lack of studies on populations outside of secondary care settings, and high risk of bias prevents us from reporting firm estimates. Future high-quality studies on the prevalence of LSS with knee or hip OA are needed.

## Supplementary Information

**Additional file 1.**

**Additional file 2.**

**Additional file 3.**

## Data Availability

The datasets used and/or analysed during the current study are available from the corresponding author on reasonable request.

## Abbreviations

LSS: Lumbar spinal stenosis
OA: Osteoarthritis
PRISMA: Preferred Reporting Items for Systematic Reviews and Meta-Analysis
