# Supplementary material for: Prevalence of multimorbid degenerative lumbar spinal stenosis with knee or hip osteoarthritis: a systematic review and meta-analysis
Source: BMC Musculoskelet Disord. 2022 Feb 24;23:177. doi: 10.1186/s12891-022-05104-3 (PMC8876450; doi:10.1186/s12891-022-05104-3)
Supplement: Supplementary file 2 — Additional file 2. [file 12891_2022_5104_MOESM2_ESM.docx]

**Additional file 2 – Search Strategy and Results**

All searches were performed on 03 May 2021.

**MEDLINE (Ovid)**

| **#** | **Searches** | **Results** |
| --- | --- | --- |
| 1 | exp Spinal Stenosis/ | 6382 |
| 2 | (spin* adj5 stenos*).mp. | 9802 |
| 3 | (lumbar adj5 stenos*).mp. | 4710 |
| 4 | (neuro* adj2 claud*).mp. | 956 |
| 5 | lumbar radicular pain.mp. | 239 |
| 6 | exp Cauda Equina/ | 3293 |
| 7 | cauda equina.mp. | 6060 |
| 8 | exp Spinal Osteophytosis/ | 4058 |
| 9 | spinal osteophytosis.mp. | 3398 |
| 10 | exp Spondylosis/ | 7767 |
| 11 | spondylos*.mp. | 5177 |
| 12 | exp Spondylolisthesis/ | 4834 |
| 13 | spondylolisthesis.mp. | 6968 |
| 14 | exp Low Back Pain/ | 22861 |
| 15 | (low* adj5 back adj5 pain).mp. | 40389 |
| 16 | 1 or 2 or 3 or 4 or 5 or 6 or 7 or 8 or 9 or 10 or 11 or 12 or 13 or 14 or 15 | 66246 |
| 17 | exp Osteoarthritis/ | 66232 |
| 18 | osteoarthr*.mp. | 100798 |
| 19 | (degenerative adj2 arthritis).mp. | 1464 |
| 20 | arthros*.mp. | 46209 |
| 21 | 17 or 18 or 19 or 20 | 140482 |
| 22 | exp Knee/ | 14766 |
| 23 | exp Knee Joint/ | 62935 |
| 24 | knee.mp. | 175832 |
| 25 | 22 or 23 or 24 | 177753 |
| 26 | exp Hip/ | 12172 |
| 27 | exp Hip Joint/ | 28142 |
| 28 | hip.mp. | 169451 |
| 29 | 26 or 27 or 28 | 169452 |
| 30 | (25 or 29) and 21 | 71219 |
| 31 | 16 and 30 | 670 |

**EMBASE (Ovid)**

| **#** | **Searches** | **Results** |
| --- | --- | --- |
| 1 | exp vertebral canal stenosis/ | 13529 |
| 2 | (spin* adj5 stenos*).mp. | 11245 |
| 3 | (lumbar adj5 stenos*).mp. | 7165 |
| 4 | (neuro* adj2 claud*).mp. | 1398 |
| 5 | lumbar radicular pain.mp. | 367 |
| 6 | exp cauda equina/ | 4742 |
| 7 | cauda equina.mp. | 9184 |
| 8 | spinal osteophytosis.mp. | 70 |
| 9 | exp spondylosis/ | 9980 |
| 10 | spondylos*.mp. | 11747 |
| 11 | exp spondylolisthesis/ | 10186 |
| 12 | spondylolisthesis.mp. | 11100 |
| 13 | exp low back pain/ | 63789 |
| 14 | (low* adj5 back adj5 pain).mp. | 74828 |
| 15 | 1 or 2 or 3 or 5 or 6 or 7 or 8 or 9 or 10 or 11 or 12 or 13 or 14 | 111736 |
| 16 | exp osteoarthritis/ | 147402 |
| 17 | osteoarthr*.mp. | 169104 |
| 18 | (degenerative adj2 arthritis).mp. | 2173 |
| 19 | arthros*.mp. | 65727 |
| 20 | 16 or 17 or 18 or 19 | 229398 |
| 21 | exp knee/ | 82399 |
| 22 | knee joint.mp. | 32436 |
| 23 | knee.mp. | 261803 |
| 24 | 21 or 22 or 23 | 261870 |
| 25 | exp hip/ | 129178 |
| 26 | hip joint.mp. | 18591 |
| 27 | hip.mp. | 252822 |
| 28 | 25 or 26 or 27 | 302389 |
| 29 | (24 or 28) and 20 | 112068 |
| 30 | 15 and 29 | 2576 |

**CENTRAL**

| **#** | **Searches** | **Results** |
| --- | --- | --- |
| 1 | MeSH descriptor: [Spinal Stenosis] explode all trees | 431 |
| 2 | spin* near/5 stenos* | 1289 |
| 3 | lumbar near/5 stenos* | 903 |
| 4 | neuro* near/2 claud* | 244 |
| 5 | lumbar radicular pain | 555 |
| 6 | MeSH descriptor: [Cauda Equina] explode all trees | 14 |
| 7 | cauda equina | 170 |
| 8 | MeSH descriptor: [Spinal Osteophytosis] explode all trees | 87 |
| 9 | spinal osteophytosis | 108 |
| 10 | MeSH descriptor: [Spondylosis] explode all trees | 382 |
| 11 | spondylosis | 936 |
| 12 | MeSH descriptor: [Spondylolisthesis] explode all trees | 221 |
| 13 | spondylolisthesis | 800 |
| 14 | MeSH descriptor: [Low Back Pain] explode all trees | 3984 |
| 15 | low* near/5 back near/5 pain | 11401 |
| 16 | #1 or #2 or #3 or #4 or #5 or #6 or #7 or #8 or #9 or #10 or #11 or #12 or #13 or #14 or #15 | 13852 |
| 17 | MeSH descriptor: [Osteoarthritis] explode all trees | 7790 |
| 18 | osteoarth* | 19529 |
| 19 | degenerative near/2 arthritis | 152 |
| 20 | arthros* | 6497 |
| 21 | #17 or #18 or #19 or #20 | 24696 |
| 22 | MeSH descriptor: [Knee] explode all trees | 822 |
| 23 | MeSH descriptor: [Knee Joint] explode all trees | 3380 |
| 24 | knee | 31703 |
| 25 | #22 or #23 or #24 | 31728 |
| 26 | MeSH descriptor: [Hip] explode all trees | 425 |
| 27 | MeSH descriptor: [Hip Joint] explode all trees | 1006 |
| 28 | hip | 24567 |
| 29 | #26 or #27 or #28 | 24567 |
| 30 | (#25 or #29) and #21 | 18007 |
| 31 | #16 and #30 | 273 |

**CINAHL**

| **#** | **Query** | **Results** |
| --- | --- | --- |
| 1 | (MH "Spinal Stenosis") | 2844 |
| 2 | TI spin* n5 stenos* OR AB spin* n5 stenos* OR TW spin* n5 stenos* | 2825 |
| 3 | TI spin* n5 stenos* OR AB spin* n5 stenos* OR TW spin* n5 stenos* | 2825 |
| 4 | TI neuro* n5 claud* OR AB neuro* n5 claud* OR TW neuro* n5 claud* | 367 |
| 5 | TI lumbar radicular pain OR AB lumbar radicular pain OR TW lumbar radicular pain | 222 |
| 6 | (MH "Cauda Equina") | 371 |
| 7 | TI cauda equina OR AB cauda equina OR TW cauda equina | 962 |
| 8 | (MH "Spinal Osteophytosis+") | 515 |
| 9 | TI spinal osteophytosis OR AB spinal osteophytosis OR TW spinal osteophytosis | 4 |
| 10 | (MH "Spondylosis+") | 2687 |
| 11 | TI spondylos* OR AB spondylos* OR TW spondylos* | 714 |
| 12 | (MH "Spondylolisthesis") | 1493 |
| 13 | TI spondylolisthesis OR AB spondylolisthesis OR TW spondylolisthesis | 1715 |
| 14 | (MH "Low Back Pain") | 20607 |
| 15 | TI low* n5 back n5 pain OR AB low* n5 back n5 pain OR TW low* n5 back n5 pain | 20085 |
| 16 | S1 OR S2 OR S3 OR S4 OR S5 OR S6 OR S7 OR S8 OR S9 OR S10 OR S11 OR S12 OR S13 OR S14 OR S15 | 33785 |
| 17 | (MH "Osteoarthritis+") | 29655 |
| 18 | TI osteoarthr* OR AB osteoarthr* OR TW osteoarthr* | 30628 |
| 19 | TI degenerative n2 arthritis OR AB degenerative n2 arthritis OR TW degenerative n2 arthritis | 348 |
| 20 | TI arthros* OR AB arthros* OR TW arthros* | 15082 |
| 21 | S17 OR S18 OR S19 OR S20 | 53825 |
| 22 | (MH "Knee") | 9654 |
| 23 | (MH "Knee Joint+") | 20223 |
| 24 | TI knee OR AB knee OR TW knee | 66186 |
| 25 | S22 OR S23 OR S24 | 72389 |
| 26 | (MH "Hip") | 6834 |
| 27 | (MH "Hip Joint") | 8641 |
| 28 | TI hip OR AB hip OR TW hip | 58232 |
| 29 | S26 OR S27 OR S28 | 61291 |
| 30 | (S25 OR S29) AND S21 | 29504 |
| 31 | S16 AND S30 | 372 |
